# Supplementary material for: Discharge care quality in hospitalised elderly patients: Extended validation of the Discharge Care Experiences Survey
Source: PLoS One. 2019 Sep 26;14(9):e0223150. doi: 10.1371/journal.pone.0223150 (PMC6762102; doi:10.1371/journal.pone.0223150)
Supplement: S1 File — (DOCX) [file pone.0223150.s001.docx]

**Supporting information**

| **File 1: Factors of the Discharge Care Experience Survey according to principal component analysis ᵃ** | | |
| --- | --- | --- |
|  |  | **Principal component**  **analysis** |
| **Factors** | **Item/ total**  **correlation** | **Loading** |
| **Coping after discharge** |  |  |
| 1. I have felt blue ᵇ | 0.70 | 0.92 |
| 2. I have felt stressed ᵇ | 0.63 | 0.87 |
| 3. I have experienced problems in getting sufficient nutrition ᵇ | 0.52 | 0.74 |
| 4. I have experienced problems in performing daily activities (e.g. personal hygiene, getting dressed or cooking) ᵇ | 0.68 | 0.63 |
| **Adherence to treatment** |  |  |
| 5. I have experienced problems in following the instructions I received when discharged from the hospital ᵇ | 0.60 | 0.88 |
| 6. I have experienced problems in understanding the instructions I received when I was discharged from hospital ᵇ | 0.58 | 0.88 |
| 7. I felt I was discharged too early ᵇ | 0.52 | 0.57 |
| **Participation in discharge planning** |  |  |
| 8. I got Information about effects and side effects of my medication | 0.60 | 0.72 |
| 9. In connection with being discharged, I had an opportunity to notify hospital personnel about what I thought was important | 0.37 | 0.77 |
| 10. When I was discharged from hospital, I had a good understanding of my responsibility in terms of looking after my health | 0.52 | 0.69 |
| 11. When I was discharged from hospital, I understood thoroughly the purpose of taking my medication | 0.53 | 0.59 |
| ᵃ Rotation method: Oblimin with Kaiser Normalization. The Kaiser-Meyer-Olkin Measure of Sampling Adequacy was 0.82. Bartlett's test of Sphericity got a P-value < 0.001, communalities varied from 0.36 to 0.80, total explained variance for the three factors were 60.4%, ᵇ Negative statements were inverted to a positive scale | | |
