# Supplementary material for: Discharge care quality in hospitalised elderly patients: Extended validation of the Discharge Care Experiences Survey
Source: PLoS One. 2019 Sep 26;14(9):e0223150. doi: 10.1371/journal.pone.0223150 (PMC6762102; doi:10.1371/journal.pone.0223150)
Supplement: S2 File — (DOCX) [file pone.0223150.s002.docx]

**Supporting information**

**File 2: Reliability analysis of the total Discharge Care Experiences Survey and its three factors**

| Scale |  | Observed data with  missing data | |  | With imputation of  missing data^a^ | |
| --- | --- | --- | --- | --- | --- | --- |
|  |  | Average  covariance | Cronbach’s  Alpha |  | Average  covariance | Cronbach’s  Alpha |
| Total (11 items) |  | 0.37 | 0.80 |  | 0.41 | 0.82 |
| Factor CAD (4 items) |  | 0.76 | 0.82 |  | 0.76 | 0.82 |
| Factor ATT (3 items) |  | 0.47 | 0.69 |  | 0.51 | 0.71 |
| Factor PiPD (4 items) |  | 0.49 | 0.64 |  | 0.52 | 0.66 |

Abbreviations: CAD=Coping after discharge; ATT=Adherence to treatment;

PiDP=Participation in discharge planning

^a^ Person mean imputation
