# Supplementary material for: Discharge care quality in hospitalised elderly patients: Extended validation of the Discharge Care Experiences Survey
Source: PLoS One. 2019 Sep 26;14(9):e0223150. doi: 10.1371/journal.pone.0223150 (PMC6762102; doi:10.1371/journal.pone.0223150)
Supplement: S3 File — (DOCX) [file pone.0223150.s003.docx]

**Supporting information**

**File 3**. Spearman correlation coefficient between the DICARES, the three factors, the NORPEQ, and other characteristics

|  | DICARES,  Total (11 items) | Factor CAD  (4 items) | Factor ATT  (3 items) | Factor PiDP  (4 items) | Patient’s  age | Charlson  Comorbidity Index | NORPEQ,  Total (6 items) |
| --- | --- | --- | --- | --- | --- | --- | --- |
| DICARES, Total (11 items) | 1 |  |  |  |  |  |  |
| Factor CAD (4 items) | 0.83^b^ | 1 |  |  |  |  |  |
| Factor ATT (3 items) | 0.73 ^b^ | 0.49 ^b^ | 1 |  |  |  |  |
| Factor PiDP (4 items) | 0.72 ^b^ | 0.38 ^b^ | 0.40 ^b^ | 1 |  |  |  |
| Patient’s age, *years* | -0.22 ^b^ | -0.20 ^b^ | -0.11 ^b^ | -0.23 | 1 |  |  |
| Charlson Comorbidity Index | -0.04 ^c^ | -0.04 ^c^ | -0.01 ^c^ | -0.02 ^c^ | -0.001 ^c^ | 1 |  |
| NORPEQ, Total (6 items) ^a^ | 0.49 ^b^ | 0.34 ^b^ | 0.40 ^b^ | 0.51 ^b^ | -0.17 ^b^ | -0.02 ^c^ | 1 |

Abbreviations: DICARES, Discharge Care Experiences Survey; CAD=Coping after discharge; ATT=Adherence to treatment;

PiDP=Participation in discharge planning; NORPEQ, Nordic Patient Experiences Questionnaire

^a^ Data was missing for two patients on the Nordic Patient Experiences Questionnaire

^b^ P value < 0.001

^c^ P value > 0.05
