# Supplementary material for: Discharge care quality in hospitalised elderly patients: Extended validation of the Discharge Care Experiences Survey
Source: PLoS One. 2019 Sep 26;14(9):e0223150. doi: 10.1371/journal.pone.0223150 (PMC6762102; doi:10.1371/journal.pone.0223150)
Supplement: Attachment — Submitted filename: PONE-D-19-15237-english.docx [file pone.0223150.s005.docx]

**Summary of research**

The publication "Discharge care quality in hospitalized elderly patients..." shows an interesting study on the extended validation of DICARES. The previously developed and validated questionnaire DICARES was subjected to an extended validation at 2 hospitals with different levels of care in Bergen (Norway) by means of a cross sectional design study. The patients were recruited from a specialized gastroenterological ward of a maximum care hospital and a general internal ward of a community hospital. The aim was to investigate the psychometric properties of DICARES and the previously identified structure of the factors. Testing was performed against other validated questionnaires.

The special feature of DICARES is its focus on accompanying the discharge ("discharge care"). The patients who were not re-admitted showed a higher DICARES score than the patients who were re-admitted. The study was conducted 30 days after discharge and the discharged patients received the questionnaire by post. The results show appropriately high values of Cronbach's alpha of the items within the factors "Coping after discharge", "Adherence to treatment" and "Participation in discharge planning" with decreasing values of 0.82, 0.71 and 0.66. The statistical tests carried out are comprehensibly documented and comprehensible.

The study, well conducted in an appropriate design and with appropriate methods, has demonstrated that DICARES is an applicable tool that can capture the quality of nursing preparation of the discharge process sensitively in terms of patient readmission and independently in terms of patient comorbidity.

The study thus proves the effectiveness of the instrument for the intended purpose.

**Overall impression**

The study meets the required ethical requirements through approval by the responsible ethics committee. The statistical analysis was carried out appropriately. An informed consent was obtained from the patients. The personal data collected was anonymized and data protection requirements for the processing and storage of the data have been fulfilled.

The study presents a well-developed and two-stage validated instrument that, with its focus on the quality of the preparation of the discharge process. The study makes an important contribution to the quality assurance of the treatment process at the interface inpatient to outpatient.

**Overall comment**

The question I am thinking about while I’m working out this peer-review is whether the fact that the questionnaire was sent 30 days after the discharge, but the readmission rate was evaluated within those 30 days, could have biased the results, and in what extent.

The questionnaires were sent at day 30 after discharge. At the same time, however, the patient readmissions were evaluated within 30 days. The study provides no information whether and how many patients who were readmitted within 30 days (especially early after discharge) got and/or completed the questionnaire.

It is correct to focus on an established hospital quality indicator such as "readmission within 30 days", as this is easy to measure. There may be patients who did not receive the questionnaire because they were already readmitted at the time the questionnaire was sent. And there may be patients who did not answer the questionnaire because they had already been re-admitted at that time, which may have resulted in a failure to answer the questionnaire even though a poor discharge process was the reason for re-admission. Despite these possible dropouts, however, the results obtained are to be regarded as correct and meaningful, as the suitability of the questionnaire was determined.

To address these thoughts, a table, graph or plot of the data showing which patients (proportions) got the questionnaire, and which answered the questionnaire depending of the status “readmitted” could be helpful.

It would be interesting to know a textual or other description of the extent to which the results differ in relation to the level of care (specialized ward / community ward).

Maybe – because this study provides a solid answer to the question to be dealt with – it would be interesting to conduct another study in which the questionnaire will be given within the first few days (e.g. 3 or 5 days) after discharge to see if the results of the questionnaire can predict the risk of a readmission.

**Overall Recommendation**

The study raises a small number of questions, some of which can be resolved by simple explanations and clarifications in the text.

Clarification should be provided on the issue of imputation and the corresponding presentation of results and textual presentation in order to remove minor ambiguities.

The planned extended validation of DICARES was carried out properly and the effectiveness and value of the instrument was proven.

The Issues to address should be worked out. The study should be published.

**Issues to address**

**Line 98-100**: The patients were recruited from a specialized gastroenterological ward of a maximum care hospital and a general internal ward of a community hospital.

> Consideration should be given in the text to whether the patient population was comparable (enough), maybe with just a few sentences. Since the wards differ in terms of specialization, the more specialized ward may have had lower readmission rates than the less specialized ward or vice versa.

Maybe consideration should be given on an adjustment of the analysis of the data depending on the level of specialization of the ward, but maybe this will be worked out in the further analysis planned as addressed in the article.

**Lines 134 ff and 155-159**: For questions addressing the imputations of data see Issues Line 201 (Table 2) and Supporting information (File 2) as follows.

**Line 201 (Table 2) and Supporting information (File 2)**: Table 2 shows information and results for calculations with imputations for missing data. The mean values with the imputations deviate in both directions (+/-) from the mean values without the imputations or remain stable. The SD sometimes gets narrower, wider or remains stable, too. Thus, it becomes clear that the imputations have an influence on the mean values shown.

> The meaning of the change in the mean values due to the imputations should be explained or presented in the text.

The total mean score is only calculated for the data without imputations.

> The total mean score in Table 2 should also be calculated for the column with the imputations.

> It should be clearly stated in the text on which mean score given in table 2 (with or without imputations) the further statements are based, as the imputations could have distorted the results.

The **File 2** provides a kind of sensitivity-analysis by calculating the average covariance and Cronbach's alpha for the data with and without imputations.

> A representation of the mean scores as in File 2 separated into data with and without imputation should be checked.

**Minor issues**

**References**: The DOI of Reference No. 28 is not correct, the article could not be retrieved via that DOI. All References should be checked, I didn’t check them systematically.

**Line 90/91**: The sentence “The psychometric…” impresses as a part of the results. The sentence should be moved to the results section or reworded.

**Line 108/109**: Only patients who completed more than 50% of the DICARES-Items were included in the study.

This criterion led to the exclusion of feedback forms that might have indicated a link between discharge preparation and readmission. The reason for the decision to set this 50% cut-off should be given in the text, as should the possible effects of this decision. Any kind of re-analysis of the data regarding this aspect seems not to be necessary.

**Line 124**: the word “patients” in the sentence “…, length of stay and patients” seems to be out of context, as long it does not address the height of the patient

**Line 176**: the percentage “53%” differs from the value given in the abstract (52%)

**Line 244/245**: There is an incomplete sentence “The concurrent validity of the.” If a complete paragraph or section is missing, I’d like to know what it was.

**Line 276**: doubled words “in order”

**Line 278**: wording should be “no correlation was found” or “no correlations were found”

**Line 283**: wording should be “reflect specific areas of discharge”

**Line 294**: wording should be “DICARES covers some similar aspects”

**Line 296**: doubled word “Factor Adherence to treatment factor was…”

**Line 340**: wording should be “The CCI is limited to only cover…” or “The CCI is limited to cover only…”

**Line 248**: wording should be “…contributes to verify…”

**Line 350**: wording should be “DICARES seems to have…”
