# Supplementary material for: Discharge care quality in hospitalised elderly patients: Extended validation of the Discharge Care Experiences Survey
Source: PLoS One. 2019 Sep 26;14(9):e0223150. doi: 10.1371/journal.pone.0223150 (PMC6762102; doi:10.1371/journal.pone.0223150)
Supplement: Attachment — Submitted filename: Response to Reviewers.docx [file pone.0223150.s006.docx]

*Discharge care quality in hospitalised elderly patients: Extended validation of the Discharge Care Experiences Survey*

We are grateful for the reviewers’ and Editor’s comments and their suggestions to improve the manuscript. We hope that our responses have addressed the comments sufficiently and that our manuscript would be acceptable for publication.

***Response to reviewers’***

The points raised by the reviewers are shown in italic, followed by our detailed response to each one of them.

Reviewers’ comments

**Response to reviewer #1**

*Overall comment

The question I am thinking about while I’m working out this peer-review is whether the fact that the questionnaire was sent 30 days after the discharge, but the readmission rate was evaluated within those 30 days, could have biased the results, and in what extent.

The questionnaires were sent at day 30 after discharge. At the same time, however, the patient readmissions were evaluated within 30 days. The study provides no information whether and how many patients who were readmitted within 30 days (especially early after discharge) got and/or completed the questionnaire.*

*It is correct to focus on an established hospital quality indicator such as "readmission within 30 days", as this is easy to measure. There may be patients who did not receive the questionnaire because they were already readmitted at the time the questionnaire was sent. And there may be patients who did not answer the questionnaire because they had already been re-admitted at that time, which may have resulted in a failure to answer the questionnaire even though a poor discharge process was the reason for re-admission. Despite these possible dropouts, however, the results obtained are to be regarded as correct and meaningful, as the suitability of the questionnaire was determined.

To address these thoughts, a table, graph or plot of the data showing which patients (proportions) got the questionnaire, and which answered the questionnaire depending of the status “readmitted” could be helpful.

It would be interesting to know a textual or other description of the extent to which the results differ in relation to the level of care (specialized ward / community ward).*

Response: Thank you. Unfortunately, we do not have permission to obtain data from patients who did not consent to participate. However, in the Discussion we have added “There may be patients who did not receive the questionnaire because they were already readmitted at the time the questionnaire was sent. Further, there may be patients who did not answer the questionnaire because they had already been readmitted at that time, which may have resulted in a failure to answer the questionnaire even though a poor discharge process was the reason for re-admission.

**Issues to address**

***Line 98-100****: The patients were recruited from a specialized gastroenterological ward of a maximum care hospital and a general internal ward of a community hospital.*

*> Consideration should be given in the text to whether the patient population was comparable (enough), maybe with just a few sentences. Since the wards differ in terms of specialization, the more specialized ward may have had lower readmission rates than the less specialized ward or vice versa.*

Response: Agree. We added information to the Methods section: “The distribution of patients with diseases of the digestive system at the specialized gastroenterology ward versus the general internal medicine ward was 48% and 18%, respectively. (line 124-126)

We also added information to the Results section: “The difference in readmission rate between the two hospital wards was insignificant (P=0.865).” (line 231-232)

*Maybe consideration should be given on an adjustment of the analysis of the data depending on the level of specialization of the ward, but maybe this will be worked out in the further analysis planned as addressed in the article.*

Response: We agree, this could be interesting to examine in future studies of DICARES.

***Lines 134 ff and 155-159****: For questions addressing the imputations of data see Issues Line 201 (Table 2) and Supporting information (File 2) as follows.*

***Line 201 (Table 2) and Supporting information (File 2)****: Table 2 shows information and results for calculations with imputations for missing data. The mean values with the imputations deviate in both directions (+/-) from the mean values without the imputations or remain stable. The SD sometimes gets narrower, wider or remains stable, too. Thus, it becomes clear that the imputations have an influence on the mean values shown.*

*> The meaning of the change in the mean values due to the imputations should be explained or presented in the text.*

Response: Agree. Sentences added to clarify: “The differences between the non-imputed and imputed data are shown in the results, and in the supporting information files. Dependent on the distribution of the responses, and the number of missing of data on each item, the mean and standard deviation may differ slightly in both directions.” (line 186-189)

We added the following text in the Discussion section: “By applying imputation the power of the analyses has been strengthen, and the risk of bias reduced. (line 411-412)

*The total mean score is only calculated for the data without imputations.*

*> The total mean score in Table 2 should also be calculated for the column with the imputations.*

Response: Agree. Total mean score for the column with the imputations have been added to Table 2. Line (line 255).

*> It should be clearly stated in the text on which mean score given in table 2 (with or without imputations) the further statements are based, as the imputations could have distorted the results.*

Response: Agree. Information added: “Cronbach’s α, **calculated using imputed data**, was estimated to be 0.82 for *Coping after discharge* (4 items), 0.71 for *Adherence to treatment* (3 items), and 0.66 for *Participation* *in discharge planning* (4 items) (S2 file).” (line 248)

**“Estimation of** Spearman’s correlation coefficient **based on imputed data,** showed a moderate relationship between the DICARES factors (S3 file),….” (line 271)

*The* ***File 2*** *provides a kind of sensitivity-analysis by calculating the average covariance and Cronbach's alpha for the data with and without imputations.*

*> A representation of the mean scores as in File 2 separated into data with and without imputation should be checked*

Response: Agree. Mean scores with and without imputations for the three factors have been added to Table 2. (line 255)

***Minor issues***

***References****: The DOI of Reference No. 28 is not correct, the article could not be retrieved via that DOI. All References should be checked, I didn’t check them systematically.*

Response: All references are checked and corrections made. We opened DOI of reference No. 28 successfully. Reference 43 (line 574-576).

***Line 90/91****: The sentence “The psychometric…” impresses as a part of the results. The sentence should be moved to the results section or reworded.*

Response: We do not agree to this point with respect to Plos One’s Submission guidelines for “Introduction”: “Conclude with a brief statement of the overall aim of the work and a comment about whether that aim was achieved”. https://journals.plos.org/plosone/s/submission-guidelines#loc-abstract

***Line 108/109****: Only patients who completed more than 50% of the DICARES-Items were included in the study.*

*This criterion led to the exclusion of feedback forms that might have indicated a link between discharge preparation and readmission. The reason for the decision to set this 50% cut-off should be given in the text, as should the possible effects of this decision. Any kind of re-analysis of the data regarding this aspect seems not to be necessary.*

Response: Agree. Text added in two sections:

“Patients completing six or more DICARES-items were included in the study, corresponding to the 50% cut-off point applied in the original version of NORPEQ”. (line 140-141)

“To obtain optimal statistical power and to retain the same number of all data **in the DICARES**, missing data in items for a person were imputed using the mean of responses of other items for that person (within person imputation), **as recommended by Siddiqui and colleagues when missing responses are ≤ 50%.”** (line 183-186)

In discussion: “Patients who completed less than six DICARES items were not included in the study. Poor condition or cognitive impairment could be reasons for lack of completion of the questionnaire. Exclusion of these patients may have biased the results”. (line 381-382)

***Line 124****: the word “patients” in the sentence “…, length of stay and patients” seems to be out of context, as long it does not address the height of the patient*

Response: Agree. Sentence reworded to: Additional information obtained from this source was age, sex, date of admission and length of stay. (line 170-171)

***Line 176****: the percentage “53%” differs from the value given in the abstract (52%)*

Response: Agree. Changed to 52%. (line 228)

***Line 244/245****: There is an incomplete sentence “The concurrent validity of the.” If a complete paragraph or section is missing, I’d like to know what it was.*

Response: Sentence removed.

***Line 276****: doubled words “in order”*

Response: Removed.

***Line 278****: wording should be “no correlation was found” or “no correlations were found”*

Response: Corrected. (line 334)

***Line 283****: wording should be “reflect specific areas of discharge”*

Response: Corrected. (line 333)

***Line 294****: wording should be “DICARES covers some similar aspects”*

Response: Corrected. (line 349-350)

***Line 296****: doubled word “Factor Adherence to treatment factor was…”*

Response: Corrected. (line 352)

***Line 340****: wording should be “The CCI is limited to only cover…” or “The CCI is limited to cover only…”*

Response: Corrected. (line 408-409)

***Line 248****: wording should be “…contributes to verify…”*

Response: Corrected. (line 417)

***Line 350****: wording should be “DICARES seems to have…”*

Response: Corrected.

**Response to reviewer #2**

*Reviewer #2: General comments:*

*- Pay attention to punctuation, for instance line 39.*
Response: Thank you. The manuscript has been checked with respect to punctuation.

*Introduction:
- The section needs major revision. The rationale for the study should be strengthened. Why is this study important to do in elderly patients? Authors must add more literature about the issue.*
Response: We agree. More text and 17 references added. (line 61 to 64, line 66-71, line 72-73, line 75-84).

*Methods:
- In general, this section should be more organized. Create some subheadings like Concurrent validity, Charlson comorbidity index and …, then provide a complete explanation for each of them.*
Response: Response: Agree. Several changes made in Methods between line 124 and line 180, several subheadings added and explanation provided:

**Concurrent validation**
“We investigated concurrent validity, a type of criterion-related validity that is suitable to measure related concepts, to examine how well DICARES correlated to two established quality indicators; the Nordic Patient Experiences Questionnaire (NORPEQ) and emergency readmission, adjusted for comorbidity”. (line 163-167)

**Charlson Comorbidity Index**

“Charlson Comorbidity Index (CCI) [16] was used to categorize comorbidity of the patients. Each comorbidity category has an associated weight (0, 1-2, 3-4 and >5), and the sum of all the weights results in a single comorbidity score for a patient”. (line 175-178)

*- In the methods section it has been stated that development and validation of the DICARES comprised literature reviews, consultations with an expert panel, patients’ evaluation and principal component analysis. This section should be enriched with additional details such as search flow and so on.*
Response: Agree. Changed made, and text added between line 146 and 161.

 *- More information is needed about the NORPEQ.*
Response: Agree. Changed to: “The survey comprised 11 DICARES items[36], and six validated items of the Nordic Patient Experiences Survey (NORPEQ)[38, 39]. NORPEQ is commonly used as a quality indicator in Norwegian hospitals and consists of eight items designed to measure patient experiences of hospital care across the Nordic countries. The six validated items assess staff interested in problem, professional skills of nurses/doctors, nursing care, understanding doctors, and information on tests. (line 134-139)

*Discussion
- Line 245, it seems to be incomplete.*
Response: We agree. Sentence removed.
