# Supplementary material for: Discharge care quality in hospitalised elderly patients: Extended validation of the Discharge Care Experiences Survey
Source: PLoS One. 2019 Sep 26;14(9):e0223150. doi: 10.1371/journal.pone.0223150 (PMC6762102; doi:10.1371/journal.pone.0223150)
Supplement: Attachment — Submitted filename: Response to Reviewers.docx [file pone.0223150.s007.docx]

*Discharge care quality in hospitalised elderly patients: Extended validation of the Discharge Care Experiences Survey*

We are grateful for the reviewers’ comments and for the opportunity to improve the manuscript.

***Response to reviewers’***

The points raised by the reviewers are shown in italic, followed by our detailed response to each one of them.

Reviewers' comments:

Reviewer #1: All my comments from the first round of the review were adequately addressed. There are only 4 minor issues left, that should be worked out prior to publication.

Line 81: missing "to", should be ...for their discharge to home
Response: Thank you, we have added “to”.

Line 123: should be ....medicine ward specialised in gastroenterology
Response: Thank you. Corrected.

Line 321: should be "In a systematic review Beattie and colleagues identified 11 instruments measuring patient experience of healthcare quality [26]." or "Beattie and colleagues identified 11 instruments of measuring patient experience of healthcare quality in a systematic review [26]."
Response: Agree. Changed to: In a systematic review Beattie and colleagues identified 11 instruments measuring patient experience of healthcare quality [26]." (line 325-326)

And - sorry for grouching, but - the DOI for Reference 43 leads me to the wrong publication, I cannot retrieve the right publication with the given DOI 10.12788/jhm.3037 via doi.org. In the citation 43 as given I can only find another publication, were the title and one authors name match.

The DOI 10.12788/jhm.3037 given in the citation 43 leads to the following publication:

Brotman DJ, Siddiqui Z, Siddiqui Z, Durkin N. Does Patient Experience Predict 30-Day Readmission? A Patient-Level Analysis of HCAHPS Data. Journal of Hospital Medicine. 2018;13. doi:10.12788/jhm.3037

The publication you cited as No. 43 has another DOI, when I look for the title only:

Siddiqui OI. Methods for Computing Missing Item Response in Psychometric Scale Construction. Current Research in Biostatistics. 2015;5: 1–6. doi:10.3844/amjbsp.2015.1.6
Response: Thank you for your thoroughness! We agree and have corrected to:
“43. Siddiqui OI. Methods for Computing Missing Item Response in Psychometric Scale Construction. Am J Biostat, 2015. doi:10.3844/amjbsp.2015.1.6” (line 578-579)

Reviewer #2: One additional suggestion to improve the manuscript content: line 147, add brief details of the search strategy.
Response: Thank you. We agree and have added: “Our search strategy comprised the following terms: *patient experience, patients satisfaction* and *patient perspective*, and further *patient discharge*, *patient transfer*, *continuity of patient care*, *patient hand over*, *patient hand* *off*, *primary health care*, *home based care*, *nursing homes*, *community health services and* and *community based care*. (line 147-151).

Line 283: wording should be “discharge”
Response: Thank you. We think this sentence was imprecise, and have added “scores on” to clarify: “The relations of scores on DICARES, and the three factors, with readmission within 30 days are shown in Table 3.” (line 287-288)

We hope that our responses have addressed the comments sufficiently and that our manuscript would be acceptable for publication.
